# Graphene-Based Electrochemical Biosensors for Breast Cancer Detection

**DOI:** 10.3390/bios13010080

**Published:** 2023-01-03

**Authors:** Ali Mohammadpour-Haratbar, Seyyed Behnam Abdollahi Boraei, Yasser Zare, Kyong Yop Rhee, Soo-Jin Park

**Affiliations:** 1 Biomaterials and Tissue Engineering Research Group, Department of Interdisciplinary Technologies, Breast Cancer Research Center, Motamed Cancer Institute, ACECR, Tehran 1715424313, Iran; 2 Department of Mechanical Engineering (BK21 Four), College of Engineering, Kyung Hee University, Yongin 17104, Republic of Korea; 3 Department of Chemistry, Inha University, Incheon 22212, Republic of Korea

**Keywords:** graphene, electrochemical biosensors, breast cancer, biomarker, nanoparticles

## Abstract

Breast cancer (BC) is the most common cancer in women, which is also the second most public cancer worldwide. When detected early, BC can be treated more easily and prevented from spreading beyond the breast. In recent years, various BC biosensor strategies have been studied, including optical, electrical, electrochemical, and mechanical biosensors. In particular, the high sensitivity and short detection time of electrochemical biosensors make them suitable for the recognition of BC biomarkers. Moreover, the sensitivity of the electrochemical biosensor can be increased by incorporating nanomaterials. In this respect, the outstanding mechanical and electrical performances of graphene have led to an increasingly intense study of graphene-based materials for BC electrochemical biosensors. Hence, the present review examines the latest advances in graphene-based electrochemical biosensors for BC biosensing. For each biosensor, the detection limit (LOD), linear range (LR), and diagnosis technique are analyzed. This is followed by a discussion of the prospects and current challenges, along with potential strategies for enhancing the performance of electrochemical biosensors.

## 1. Introduction

Cancer presently ranks among the deadliest diseases in the world [1,2,3]. In particular, breast cancer (BC) is the most common reason of cancer-related death [4,5]. Moreover, while women have always been prone to the disease [6], the incidence of BC is increasing globally, and is higher among women living in less developed countries [7]. Because the chances of successful cancer treatment are greatly increased by early detection, there is widespread interest in early cancer diagnosis [8]. Hence, it is crucial to detect cancer at an early stage by identifying cancer cells [9,10].

A particularly effective technique for the early diagnose of BC based on the detection and quantification of BC biomarkers is provided by an electronic device called a biosensor [11,12,13,14]. This typically consists of the following main components: (i) a bio-receptor, which is a biomolecule that recognizes a target analyte and reacts with it physically or chemically, and (ii) a transducer, which converts recognition into a measurable signal [15,16]. A biosensor can be electrical, electrochemical, optical, or mechanical, according to the type of measurable signal [17]. Among these, the electrochemical biosensors are particularly attractive because of their great sensitivity and selectivity, simplicity, and low price [18,19,20,21]. Hence, designing an electrochemical biosensor for BC detection can be very helpful for patients suffering from the disease [22] The detecting performance of a biosensor is assessed by calculating the linear range (LR), sensitivity, response time, limit of detection (LOD), selectivity, stability, and repeatability.

As a two-dimensional (2D) nanomaterial, graphene (Gr) has contributed greatly to the electronic and biosensor industries [23,24,25]. The term graphene refers to a sheet of hexagonally arranged, sp^2^-bonded carbon atoms that occurs within a carbon material [26,27,28]. Graphene is known for its excellent electrical conductivity. In the literature, graphene-based nanomaterials are used in biosensors to detect biomarkers, cells, DNA, glucose, pH, and other analytes. In electrochemical sensors fabricated with graphene-based materials, electron transfer occurs rapidly at edges and defects compared to the basal planes. The presence of these structural defects in chemically modified graphene can be exploited for electrochemical sensor applications [29,30]. The large theoretical surface area of graphene as well as its superior electrical conductivity make it an ideal electrode material. Additionally, graphene exhibits remarkable electrochemical properties, including large potential windows, low charge-transfer resistance, high electrochemical activity, and fast electron transfer [31,32]. Other influential features of graphene are two-dimensionality, limited size, robustness, and the possibility of mixing carbon-based materials with different features guaranteeing greater integration. Therefore, graphene is an excellent material for electrochemical sensing due to its excellent physical and electrochemical properties. Due to their biocompatibility, outstanding conductivity, and exceptional mechanical performances, the graphene family, including reduced graphene oxide (rGO), graphene oxide (GO), and graphene quantum dots (GQDs), are extensively used in the fabrication of biosensors, drug delivery systems, and energy storage devices [33,34,35,36]. A graphene-based material containing oxygen-containing functional groups is crucial to electrochemical sensors, as they enable adsorption and preconcentration of redox species (which are of interest to us analytically) and catalyze redox reactions effectively. In addition, the presence of these functional groups makes an effective functionalization with various biomolecules and polymers for applications [37,38]. Actually, the use of graphene-based electrochemical biosensors for cancer detection is mainly due to their ability to absorb several molecules and bind easily with them under certain conditions. The functionalization of these graphene-based materials with specific functional groups can enable the use of these excellent materials for electrochemical sensor applications with specific analytes. Moreover, these nanomaterials exhibit a great surface area, thereby improving the sensitivity of the biosensor by increasing the level of bioreceptor immobilization [39,40].

The present review presents a compressive overview of the various electrochemical approaches available for the biosensing of BC biomarkers, with a particular emphasis on the graphene materials. The analytical performance of each biosensor, especially the LR and LOD, are discussed, and the final section discusses the challenges and successes of graphene-based biosensors for BC sensing.

## 2. Biomarkers of BC

**Figure 1:**
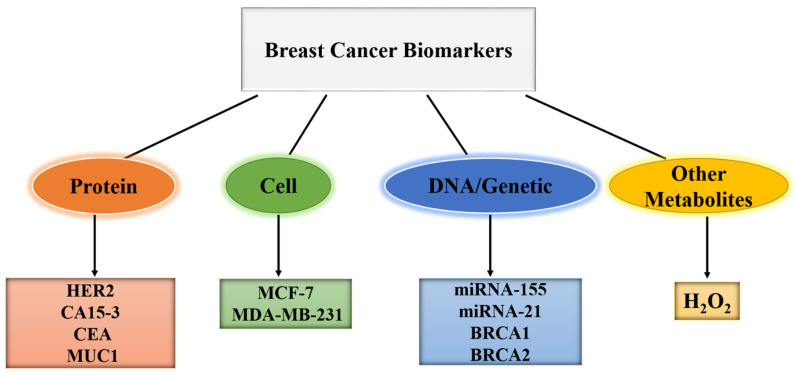
Types of biomarkers associated with BC.

A biomarker is an objective measurement and evaluation characteristic that can be used to determine whether an organism or pathogenic process is normal, or if therapeutic intervention is required [41,42,43]. In other words, biomarkers are chemical indicators of disease status, which can be used to differentiate between a cancerous and a normal tumor [44,45]. Thus, biomarkers provide information about how cancer develops and progresses in the body. Blood, urine, saliva, and other body fluids all contain biomarkers, and can be used as analytes in sensor development [46,47,48]. As shown in Figure 1, examples of BC biomarkers include protein biomarkers such as human epidermal growth factor receptor 2 (HER2), Carcinoembryonic Antigen (CEA), Cancer antigen 15-3 (CA15-3), Mucin 1 (MUC1), cell biomarkers such as Michigan cancer foundation-7 (MCF-7), MDA-MB-231, DNA/genetic biomarkers such as microRNA-155 (miRNA-155), microRNA 21 (miRNA-21), breast cancer 1 (BRCA1), breast cancer 2 (BRCA2), cluster of differentiation 24 (CD-24), cluster of differentiation 44 (CD-44), and other metabolites such as H_2_O_2_ [49,50,51,52].

## 3. Graphene and Its Derivatives for the Electrochemical Sensing of BC Biomarkers

Due to the growing number of BC patients, electrochemical biosensors have received increased consideration for the detection of BC biomarkers [53,54,55]. In this respect, the high surface area, suitable mechanical properties, and outstanding conductivity of graphene and its derivatives have led to the improvement of biosensor performance, thus making them important components of electrochemical biosensors for BC detection [56,57,58]. Graphene derivatives can differ from pristine graphene in terms of their properties. For example, there is a significant difference between pristine graphene, which has a honeycomb lattice structure, and thermally reduced graphene oxide (TRGO), which has a large number of defects [59]. Defects are not detrimental. Contrary to popular belief, in the electrochemistry of sp2 carbons, heterogeneous electron transfer occurs at the edges and defects, not at the basal plane [60]. On the other hand, GO has a damaged sp2 carbon network, which makes its structure not entirely planar. It has a large amount of oxygen-containing groups, which can be beneficial to the functionalization through biomolecules during biosensing [61]. Hence, the features of pristine graphene and its derivatives (GO, rGO, and GQD) are examined in the following subsections, along with those of graphene-based electrochemical biosensors for BC biomarker detection.

### 3.1. Electrochemical Biosensors Based on Pristine Graphene

**Figure 2:**
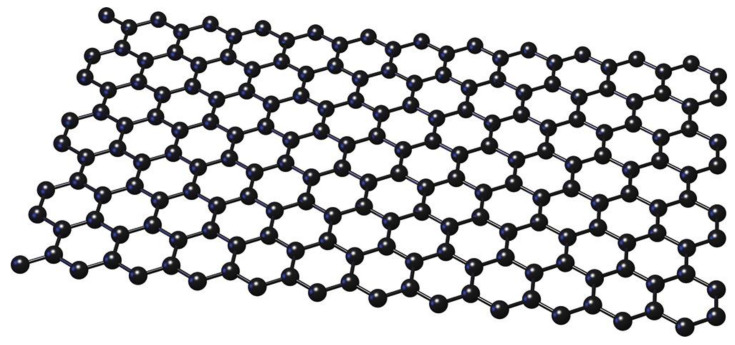
The structure of pristine graphene.

Since its discovery in 2004, graphene has revolutionized a variety of scientific fields such as electronics, nanocomposites, and biosensing [62,63]. The construction of pristine graphene is shown in Figure 2. Its great conductivity, excellent strength, and big surface area have made graphene a hot topic in the last few years. It is among the ideal materials for application to electrochemical biosensor electrodes [64,65], providing an increase in both sensing and electron transferring. Table 1 summarizes recent developments in graphene-based electrochemical biosensors for the diagnosis of BC biomarkers.

The microRNAs (miRNAs) play a role in many biochemical processes [66], and are released by various kinds of malignant cells. Hence, a cancer type can be classified based on the irregular expression of a specific miRNA [67]. Moreover, researchers have found that high miRNA levels are correlated with various types of cancer [68,69]. Thus, the miRNAs play key roles as biomarkers in early cancer detection, cancer type identification, cancer stage estimation, and cancer treatment monitoring [70]. In particular, miRNA levels are of crucial importance to improving the prognoses in early-stage cancer detection via ultra-trace analysis. Indeed, one of the most promising biomarkers for the diagnosis, prognosis, and therapy of BC is miRNA-21 [71].

**Table 1:** Electrochemical biosensors of graphene-based materials for BC detecting.

Electrode	Detection Technique	Target	LR	LOD	Ref.
Gr/poly-L-lysine	DPV	miRNA	-	1 fM	[72]
Gr foam/TiO_2_ nanofibers	EIS	ErbB_2_	1 fM–0.1 μM	-	[73]
Gr/Anti-CA 15-3/GCE	DPV	CA 15-3	0.1–20 U/mL	0.012 U/mL	[74]
Gr/AuNPs/PPY	DPV	miRNA-21	1 fM–1 nM	0.02 fM	[75]
Gr/Herceptin/GCE	DPV	HER2	-	-	[6]
Gr/DNA/AuNPs/GCE	CA	BRCA1	1 fM–1 nM	1 fM	[76]
N_2_-doped Gr/AgNPs/PANI ^a^	DPV	HER2	10–5 × 10^6^ cells.mL^−1^	2 cells.mL^−1^	[77]
N_2_-doped Gr/AgNPs/PANI	DPV	miRNA-21	10 fM–10 μM	0.2 fM	[78]
3DGrH/AuNPs	DPV	CA 15-3	10^–2^–150 U.mL^–1^	11.2 × 10^–2^ U.mL^–1^	[79]
Gr/Au nanorods/GCE	DPV	CEA	5 pg.mL^−1^–50 ng.mL^−1^	1.5 pg.mL^−1^	[80]
Gr aerogels/SIL	DPV	BRCA1	-	3 pM	[81]
Gr/meso-SiO_2_/PET ^b^	CA	HER2	-	0.6 × 10^−15^ M	[82]
amine-functionalized Gr/GCE	DPV	miRNA-155	3 × 10^−11^–10^−9^ M	1.25 × 10^−11^ M	[83]
Gr/dNCs ^c^	CA	H_2_O_2_ from MCF 7	1 pM–10 μM	1 pM	[84]
Gr/AuNCs ^d^/MWCNTs ^e^/ Ab1 ^f^/GCE	DPV	MCF 7 cells	10^2^–10^6^ cells.mL^−1^	80 cells.mL^−1^	[85]

a: polyaniline, b: polyethylene terephthalate, c: dispersed nanocavities, d: Au nanocages, e: multiwalled carbon nanotubes, f: antibodies.

**Figure 3:**
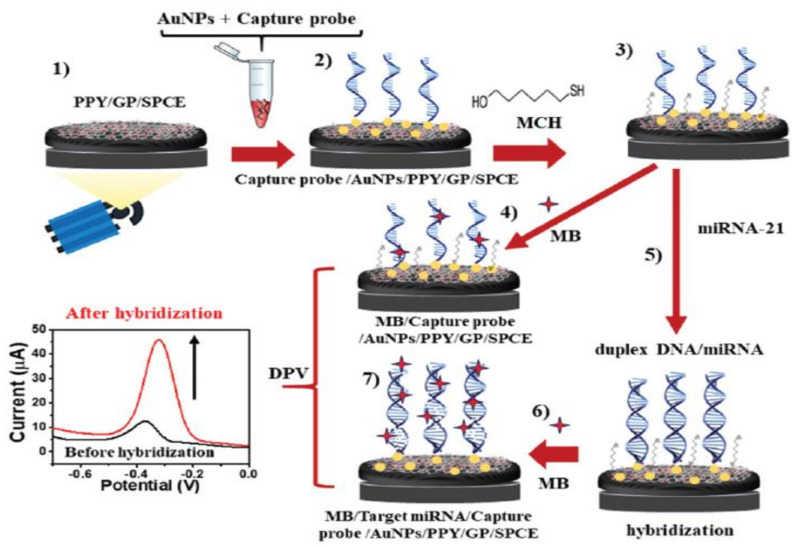
A schematic diagram showing the various steps in the fabrication of a biosensor. Reprinted from Ref. [75], with permission from RSC.

Many studies have reported that graphene can be used as a biosensor material for detecting BC biomarkers. For example, Pothipor et al. [75] designed a biosensor using graphene, polypyrrole (PPY), and gold (AuNPs) for the diagnosis of miRNA-21. The modification of a carbon electrode (SPCE), and the preparation of the biosensor are revealed schematically in Figure 3. Both the surface and conductivity of the electrode were shown to be boosted by graphene. Furthermore, the addition of PPY was shown to grow the effective surface of the electrode, and to improve the dispersibility of the AuNPs on the surface. This is advantageous because AuNPs can enhance the immobilization of DNA probes due to their good biocompatibility and high dispersibility. AuNPs, as a kind of inorganic nanoparticle, have gradually been recognized as promising nanomaterials due to their unique optical, electronic, sensing, and biochemical properties. As a result of their unique properties, AuNPs have found wide application in biomedical fields including diagnosis, biosensing, and drug delivery [86]. AuNPs can also increase electron movement in biosensors due to their high conductivity. As a result, the biosensor exhibited LR values in the range of 1 fM to 1 nM, and an LOD of 0.02 fM.

Breast cancer 1 (BRCA1) is a human caretaker gene found in breast and other tissues [87]. Several hundred mutations in the BRCA1 gene that are associated with an increased risk of BC have been identified [88,89]. Therefore, the diagnosis of BRCA1 gene mutation in the early stages can be very beneficial for BC patients. Hence, a biosensor based on graphene for the diagnosis of the BRCA1 biomarker was developed by Abdul Rasheed et al. [76]. The biosensor presented high stability and sensitivity to the BRCA1 biomarker, with an LOD of 1 fM and an LR ranging from 1 fM to 1 nM. In another study, Kazerooni et al. [81] presented a biosensor for the diagnosis of BRCA1 biomarkers by using supramolecular ionic liquids (SILs) and graphene aerogels. BRCA1 DNA was directly detected using SILs grafted onto graphene aerogel-modified glassy carbon electrode (GCE). There are several advantages to a graphene nanocomposite functionalized with SIL, including high dispersion, functionality, high specific surface area, and high surface charge density [90]. Graphene aerogels have great strength-to-weight ratios and large surface areas [91], along with porous three-dimensional (3D) frameworks that can provide multidimensional electron transport pathways, thereby increasing the performance of the biosensor [92]. Meanwhile, the high surface charge density of the SIL results in enhanced interaction with the biomarkers [90]. As a result, the biosensor exhibited an LOD of 3 pM.

Carcinoembryonic antigen (CEA) is a broad-spectrum biomarker that is elevated in many types of cancer, including gastric, breast, liver, and pancreatic cancers [93]. Positive CEA levels are associated with disease progression or regression status, and their levels are used to monitor treatment effectiveness and to detect recurrences early [94,95]. Biosensors have attracted much attention as a method for detecting CEA biomarkers. Hence, a biosensor for the diagnosis of CEA biomarkers was constructed by Wen et al. [80] using a graphene nanocomposite and Au nanorods. They demonstrated that the effective surface area of the electrode and, hence, the biosensor sensitivity were greatly increased by the presence of graphene, due to its intrinsically great surface area. Additionally, the superior conductivity, edge sites, and high electron transfer rate of graphene significantly increased the transfer of electroactive agents at the electrode surface. Consequently, the biosensor exhibited a low LOD of 1.5 pg mL^−1^ toward CEA detection and LR ranging from 5 pg mL^–1^ to 50 ng mL^–1^.

### 3.2. Electrochemical Biosensors Based on Graphene Oxide (GO)

**Figure 4:**
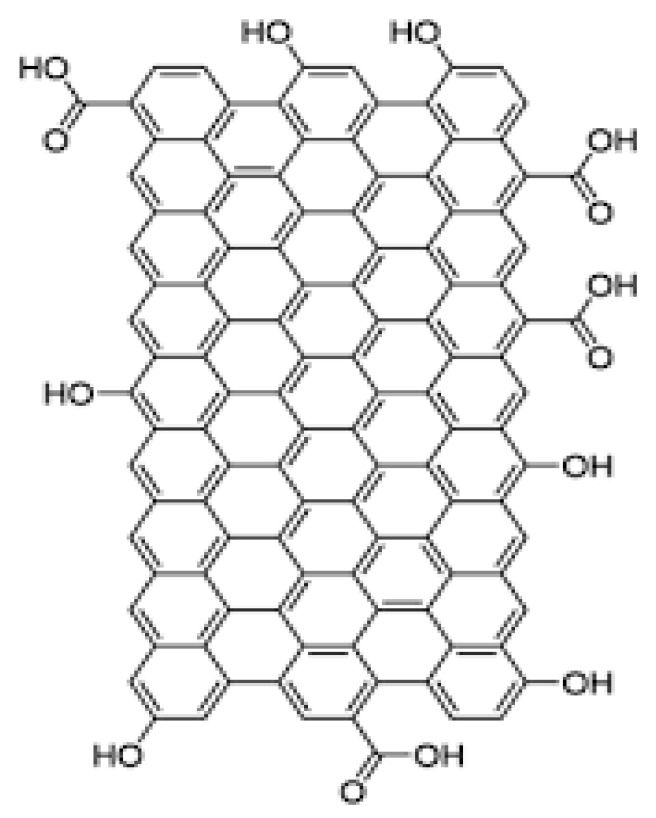
The structure of GO.

Although graphene is relatively novel, GO was shown in the initial studies on the graphite chemistry [61], synthesized by the chemical exfoliation of oxidized graphite [96]. A variety of properties make GO an ideal material for use in biosensors, including its high specific surface area, extensive functionality, excellent stiffness, good electron transportation, and outstanding biocompatibility [97,98,99]. The structure of GO is presented in Figure 4. Due to its oxygen groups, GO can provide an ideal substrate for immobilizing bioreceptors on its surface [100,101]. GO-based biosensors for detecting BC are summarized in Table 2.

The use of GO in the construction of biosensors for detecting BC biomarkers has been reported by many groups. Recently, Pothipor et al. [102] developed an electrochemical biosensor for the concurrent diagnosis of cancer antigen 15-3 (CA 15-3) and miRNA-21 by modifying an SPCE with a GO/poly(3-aminobenzylamine)/molybdenum selenide (MoSe_2_)/AuNPs nanocomposite. MoSe_2_ is an excellent material for sensing applications due to its high specific surface area and fast charge transfer [115]. The peak current of the biosensor was found to increase when the SPCE was coated with 2D-MoSe_2_ nanosheets, thereby indicating that the selenium in the 2D-MoSe_2_ structure is intrinsically conductive [115]. Meanwhile, the presence of the conducting polymer, poly(3-aminobenzoic acid) (P3ABA), in the nanocomposite was shown to enhance the electrochemical reactivity and compatibility, thereby making the biosensor more sensitive, and providing faster electron transfer kinetics. On the other hand, the high conductivity and biocompatibility of AuNPs make them ideal for use in the biosensor field because they provide uniform electroactive sites and large surfaces for immobilizing biomolecules [116]. Consequently, the as-developed biosensor exhibited an appropriate LR for the concentrations of both analytes, with the LODs of 0.14 U mL^–1^ for CA 15-3, and 1.2 fM miRNA-21.

Various types of cancer are caused by abnormal glycosylation and overexpression of MUC1, a transmembrane glycoprotein found on the apical surface of epithelial cells [117,118,119]. A high concentration of MUC1 in the early stages of BC can be used as a diagnostic biomarker [120,121]. Various electrochemical biosensors have been investigated for detecting this BC biomarker [119,122,123]. For instance, Bharti et al. [105] produced a biosensor for the analysis of MUC1 by modifying the surface of a fluorine-doped tin oxide (FTO) electrode with a compound of gold platinum bimetallic nanoparticles (Au-PtBNPs) and carboxylated graphene oxide (CGO). GO nanosheets with -COOH modifications provide a better platform for immobilizing biomolecules. On the other hand, the sensitivity of biosensors can also be increased using bimetallic nanoparticles. Compared to monometallic nanoparticles, the synergistic effect of bimetallic nanoparticles exhibits unique optical, catalytic, and electrochemical properties [124]. The presence of Au-PtBNPs was shown to improve the conductivity of the electrode, and the resulting biosensor had an excellent discrimination of the MUC1 biomarker, with a small LOD of 0.79 fM, and an extensive LR from 1 fM to 100 nM. In another study, Gupta et al. [107] designed a biosensor for the MUC1 biomarker with a conducting polymer nanocomposite of AuNPs, GO, and poly(3,4-ethylenedioxythiophene) (PEDOT). The high electrical conductivity and superior electrochemical stability of PEDOT make it an attractive sensor platform due to its biocompatibility [125,126]. They found that the PEDOT considerably enhanced the electrochemical properties of the nanocomposite, while the AuNPs and GO enhanced the electroactive surface area of the AuNPs-GO-PEDOT electrode, thereby improving the electrochemical sensing performance. As a result, the AuNPs-GO-PEDOT provided a highly sensitive platform for measuring MUC1, with an LOD of 0.031 fM, and an LR of 3.13 aM to 31.25 nM.

**Figure 5:**
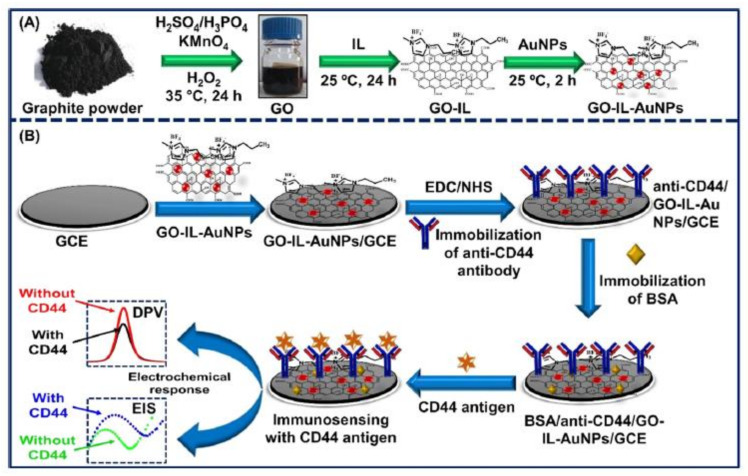
A schematic design of (**A**) the production of the GO-IL-AuNPs composite, and (**B**) the step-wise construction of the biosensor. Republished from Ref. [114], with permission from ACS.

The differentiation-44 (CD44) antigen is a transmembrane glycoprotein found on cell surfaces [127]. Several studies have shown that CD44 is expressed on the surface of cancer stem cells, which may contribute to cancer beginning and development. Numerous cancers are associated with high CD44 concentrations, including breast, prostate, lung, colon, etc. [128,129]. Moreover, CD44 has been found to exhibit metastatic cancerous behavior in clinical studies, thereby suggesting that it can serve as a BC biomarker. Therefore, the CD44 antigen could serve as a key early-stage biomarker for investigating BC growth [130,131]. Hence, Ranjan et al. [114] used a nanocomposite of GO, Au NPs, and an ionic liquid (IL) to develop a biosensor for CD44 detection, as shown schematically in Figure 5. ILs possess excellent physical, chemical, and electrochemical properties, such as high conductivity, a wide electrochemical window, low volatility, high stability, high stabilization power, and strong adhesive properties. Furthermore, they possess excellent biocompatibility and stabilizing properties for biomolecules [132,133]. Numerous studies suggest that the combination of ILs and GO produces better material than either ILs or GOs alone [134,135]. The GO was shown to facilitate the immobilization of antibodies due to its oxygen-containing functionalities, while the AuNPs enhanced the performance of the biosensors by facilitating charge transfer and growing the effective surface area. The LR of the resulting CD44 biosensor ranged from 5 fg mL^−1^ to 50 μg mL^−1^, with LOD = 2 fg mL^−1^.

### 3.3. Electrochemical Biosensors Based on Reduced Graphene Oxide (rGO)

**Figure 6:**
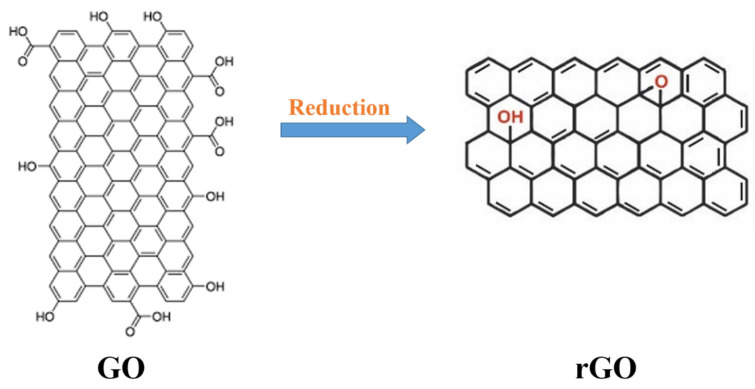
The reduction of GO to rGO, and the chemical structure of rGO.

An attractive feature of GO is its ability to reduce to graphene-like nanosheets when the oxygen-including groups are removed, and the conjugated structures are recovered. An rGO sheet is typically referred to as a chemically-derived graphene [136]. A reduction procedure provides the graphene-like nanosheets with similar characteristics and structures to those of the pristine graphene gained by mechanical exfoliation of graphite layers. The reduction process of GO and the chemical building of rGO are exposed in Figure 6, where the rGO surface is seen to have fewer functional groups. Recently, a great deal of interest has been focused on rGO as a 2D carbonaceous material with a great surface area, excellent conductivity, and high carrier mobility [137]. Moreover, the reduced chemical groups make it a potential biosensing material because of its ultra-high stability and ease of functionalization [138]. Table 3 summarizes the biosensors of rGO for BC biomarkers.

Many teams have reported the usage of rGO and its nanocomposites as biosensor materials for the detection of BC biomarkers. For example, measurement of the biomarker human epidermal growth factor receptor 2 (HER2) is applied as a clinical test for the detection of BC [162]. It has been found that HER2 attention in the blood of a healthy person varies from 2 to 15 ng mL^–1^, while that in the cancer patient blood changes from 15 to 75 ng mL^–1^ [163]. Consequently, the development of low-cost, fast, and accurate biosensors for the diagnosis of HER2 is crucial to the early detection of cancer, the monitoring of treatment effectiveness, and the assessment of remission risk after treatment. For example, Forootan et al. [139] modified the GCE with single-walled carbon nanotubes (SWCNTs), rGO, and AuNPs to fabricate a biosensor capable of detecting the HER2 biomarker. Their results demonstrated that the rGO and AuNPs were capable of enhancing the electron transfer and available surface on the GCE, thereby increasing the current response of the biosensor compared to that of bare GO. This is due to the great surface and exceptional conductivity of rGO, and to the remaining oxygen-related defects. The resulting biosensor exhibited an LR of between 0.1 pg mL^–1^ and 1 ng mL^–1^, and an LOD = 0.05 pg mL^–1^. In another study, Augustine et al. [142] presented a HER2 biosensor with excellent selectivity and sensitivity using molybdenum trioxide (MoO_3_) anchored onto rGO, and found that rGO nanosheets with edgelike defects accelerated the charge transfer at the electrode–solution interface, thereby acting as electron promoters. MoO3 exhibits a number of promising properties, including its versatile chemical and physical properties, its variable oxidation state, a high catalytic activity, an extremely wide band gap, great electrical properties, as well as a high level of biocompatibility [164,165]. Additionally, polarity and variable oxidation states increase affinity for biomolecules, resulting in faster electron transfer, leading to improved sensitivity. Moreover, the high aspect ratio of the MoO_3_ nanorods further increased the loading capacity of biomolecules, thus allowing for better interactions between the electrode surface and the goal analyte. The offered biosensor displayed a wide LR of 0.001−500 ng mL^−1^, along with an LOD of 0.001 ng mL^−1^.

Carbohydrate antigen 15-3 (CA15-3) plays a vital role as a biomarker in the diagnosis of BC. A healthy individual has a serum CA15-3 concentration of less than 30 U mL^–1^, whereas levels higher than 100 U mL^–1^ can show the presence of BC [166]. Moreover, the CA15-3 concentration is a highly valuable indicator of treatment success. Consequently, the sensitive and selective detection of the CA15-3 biomarker is highly crucial for clinical diagnosis [51]. For example, an electrochemical biosensor for the diagnosis of CA15-3 was developed by Amani et al. [143], applying a screen-printed electrode (SPE) altered with a CuS-rGO composite. The exceptional optical, electronic, physical, and chemical properties of CuS have gained significant attention over the past few years, which revealed their potential application as electrochemical catalysts [167]. CuS nanoparticles supported on RGO sheets were much more dispersible than CuS alone, resulting in greater catalytic activity. CuS nanoparticles agglomerated severely when RGO was absent. This is due to the high surface-to-volume ratio of CuS nanoparticles, which causes them to self-agglomerate during re-dispersion in water.

**Figure 7:**
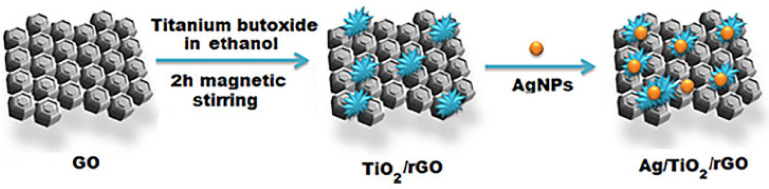
Schematic synthesis of an Ag/TiO_2_/rGO nanocomposite. Republished from Ref. [159], with permission from Elsevier.

The electron transfer resistance (R_ct_) of the CuS/rGO/SPE was determined by measuring the diameter of the semicircle in the impedance spectrum, and was lower than that of the rGO/SPE and the bare SPE due to the superior electrical features of the CuS/rGO nanocomposites, which resulted in a large electron conduction pathway. In another work, Shawky et al. [159] produced a signal amplifier platform based on a silver/titanium dioxide/rGO (Ag/TiO_2_/rGO) ternary nanocomposite for the fabrication of an electrochemical biosensor. The synthetic process is shown schematically in Figure 7. The chemical stability, low toxicity, and high activity of TiO_2_ have made it an attractive biosensor material [168]. As a result of the electrocatalytic action of the ternary Ag/TiO_2_/rGO composite, the as-prepared biosensor had an ultrahigh electrochemical sensitivity and superior activity toward the quantification of the BC antigen CA 15-3, with LOD = 0.07 U mL^–1^ and LR of 0.1 to 300 U mL^–1^.

Many cancer cells are typified by the excessive presence of reactive oxygen species (ROS) as compared to their healthy counterparts [169,170]. In particular, hydrogen peroxide (H_2_O_2_) is a vital ROS that is associated with many physiological and pathological processes, and can cause numerous illnesses such as cancer, Alzheimer’s, heart attacks, and Parkinson’s disease by damaging the proteins, lipids, and DNA within cells [171,172]. Furthermore, since cancer cells generate more H_2_O_2_ than normal cells, H_2_O_2_ can act as a biomarker for detecting cancer cells [173]. Hence, it is crucial to detect H_2_O_2_ effectively and accurately in living cells in order to monitor its concentration and understand the relevant biological processes. For example, Dong et al. [148] presented a biosensor for the diagnosis of H_2_O_2_ by modifying a GCE with Au-Pd nanocube nanocomposites that were assembled on rGO, and demonstrated that the resulting adjustment of the electrode surface with 3D nanostructures, and the increased attendance of active sites, greatly improved the electrocatalytic H_2_O_2_ reduction performance of the electrode. The latter effect was qualified to the huge electroactive surface and superior electrical conductivity of the 3D nanocomposites. The resulting biosensor exhibited a low LOD of 4 nM, and a wide LR ranging from 0.005 μM to 3.5 mM. Similarly, Li et al. [160] prepared a biosensor of H_2_O_2_ by modifying a GCE with ZnMn_2_O_4_/rGO-wrapped rGO microspheres to provide a high surface area and effective conduction channels, thereby increasing both the conductivity and H_2_O_2_ reduction ability. Due to the synergistic effects of the ZnMn_2_O_4_ and rGO, the resulting ZnMn_2_O_4_/rGO-based biosensor exhibited a wide LR of 0.03−6000 μM, and an LOD of 0.012 μM.

### 3.4. Electrochemical Biosensors Based on Graphene Quantum Dot (GQD)

**Figure 8:**
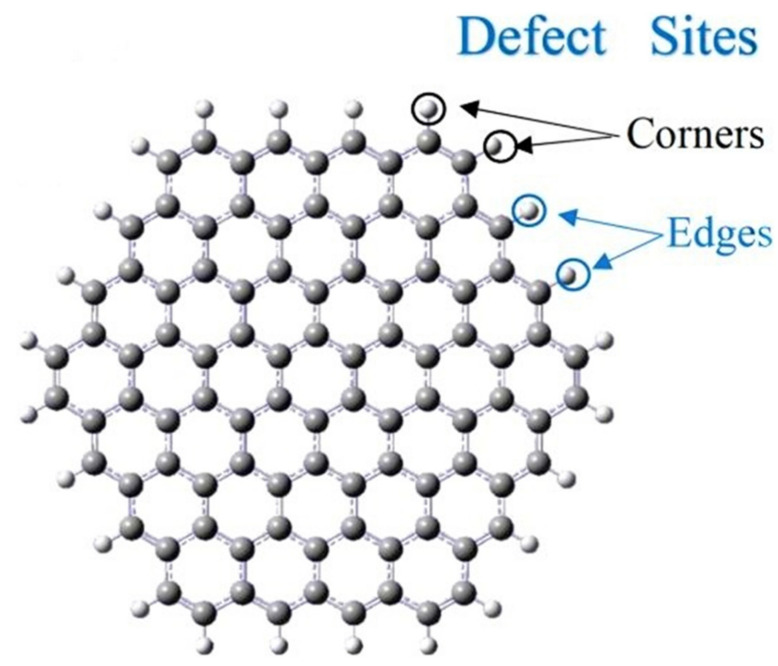
Structure of GQD.

Quantum dots (QDs) are inorganic semiconductors with unique optical and electronic properties, and have generated significant interest over the past two decades [174,175]. In particular, GQDs are small graphene fragments in which electronic transport can occur in three spatial dimensions [176]. The chemical building of the GQD is exposed in Figure 8. The special edge and quantum confinement effects of the GQDs distinguish them from other carbon materials such as CNTs, carbon QDs, and graphene [177,178]. The GQDs usually have a size range of below 20 nm and excellent chemical, physical, and biological properties [179,180]. The small size of GQD makes it a better candidate for biomedical applications than graphene and graphene oxide [181]. GQDs are used in numerous applications, including drug delivery systems, bioimaging, and biosensors [180,182,183]. GQD-based electrochemical biosensors have gained considerable attention in recent years for the detection of various analytes. A summary of GQD-based biosensors for the diagnosis of BC is provided in Table 4.

The attention of MCF-7 is another key biomarker for the early identification of BC, as a luminal-A subtype of the BC cell line. Hence, Tran et al. [184] designed a biosensor for the diagnosis of MCF-7 by depositing nitrogen-doped GQDs (NGQDs) and phytohemagglutinin-L (PHA-L) onto SPEs. Their results demonstrated that the NGQDs helped as nanocarriers for PHA-L and electronic conductors, thereby providing a superior electrochemical sensing performance. Accordingly, the biosensor displayed a linear range of 5–10^6^ cells mL^–1^ and a low LOD of 1 cell mL^–1^. Meanwhile, Hasanzadeh et al. [185] developed a biosensor by using a nanocomposite containing gold nano-shrubs (Au NSs), GQDs, and cysteamine (CysA) for the diagnosis of the CA 15.3 biomarker. A new method of enhancing the conductivity of graphene-based nanomaterials is the fabrication of Au NSs in graphene-based materials. This method is an excellent way of preparing a highly electrically performing organic–inorganic nano-hybrid with nanoparticles incorporated into it. The electron transfer efficiency of the resulting biosensor was effectively improved by the synergistic interaction between the GQDs-CysA as conductive substrate and the Au NSs as signal amplifiers. Moreover, the GQDs-CysA offered a high surface area, thereby allowing the target analyte to be further immobilized. The biosensor exhibited LRs from 0.3–1 U/mL to 2–250 U/mL, along with a LOD = 0.011 U/mL.

## 4. Summary and Future Trends

Researchers have been developing biosensors for detecting biomarkers of BC in recent years. Nanotechnology and new technologies have made biosensors an indispensable and useful tool for detecting BC. Detecting BC biomarkers using electrochemical biosensors has made remarkable progress in recent years due to nanotechnology and biosensor techniques. The present review has discussed the recent advancement in graphene-based biosensors, including the design strategies and results for the sensing of BC cells. The inherent features of graphene and its family were discussed, along with their applications in the biosensors of BC cells. Graphene oxide (GO) nanosheets modified with -COOH can be used to immobilize biomolecules more efficiently. Its functionalized groups make GO ideal for immobilizing bioreceptors on its surface. As compared to pristine graphene, reduced graphene oxide (rGO) contains a large number of defects. In contrast to popular belief, heterogeneous electron transfer occurs at the edges and defects of sp2 carbons, not at the basal plane. There is a wide variety of uses for rGO, such as drug delivery systems, reinforcing nanocomposites, increasing electrical properties, and especially as biosensors for measuring cancer biomarkers. Graphene quantum dots (GQDs) are new graphene-based nanomaterials used in a wide range of applications, including drug delivery, bioimaging, and biosensors. The development of GQD-based electrochemical biosensors to detect various analytes has received considerable attention in recent years. Numerous graphene-based nanomaterials were developed for use in biosensor devices, while probes such as DNA, antibodies, aptamers, proteins, or small organic molecules are used to recognize a selected target.

In order to commercialize the developed biosensor for detection of BC biomarkers, the main challenges must be overcome first. While many graphene-based biosensors have been reported as stable and repeatable, other biosensors fail to achieve the desired detection results when used with real samples. This is mainly because the interfacial reactions between the graphene and various biological and chemical molecules are non-specific towards the desired targets. Moreover, the preparation of biological samples is necessary before the final tests, and involves potentially time-consuming and complicated procedures such as separation or preconcentration. On the other hand, dispersing graphene nanoparticles in other materials can be challenging and requires functionalizing the graphene surface, which increases the cost of biosensor manufacture. BC detection can be greatly improved by the discovery of specific and sensitive biomarkers. In spite of this, there has been little progress in the identification of new biomarkers and the development of highly sensitive and reproducible biosensors for early BC detection. Additionally, commercializing R&D technology may require more time and funding. In spite of several publications, developing substrates that are highly specific to BC biomarkers can be a challenge since most biomarkers are also found in other cancers. Therefore, developing strategies to simultaneously detect multiple biomarkers and avoid false positives is another important challenge for electrochemical biosensors. Because of the low concentration of most BC biomarkers, it is also possible that BC biosensors do not cover the whole range of biomarker concentrations. Therefore, continuous efforts are needed to overcome these challenges in order to produce large-scale graphene-based biosensors. Currently, efforts are being made to develop novel biosensors to detect BC cells to address these challenges. Ultimately, this study will aid researchers in the development of biosensors that can detect BC biomarkers in the early stages and improve cancer prognosis and treatment.

The graphene-based nanomaterials may become a base for future contributions. For example, it could be used in a wide range of biosensor applications for early and faster diagnosis. Indeed, they could be used in liquid biopsy by detecting Circulating Tumor DNA (ctDNA) in a patient’s blood. In a new study, researchers from the University of Illinois at Urbana-Champaign found that “crumpled” graphene makes biosensors extremely more sensitive to DNA [188]. Moreover, a 3D porous graphene metal nanocomposite is also used for glucose and pH sensors. The potential of this novel fabrication technique and the extensible metal nanocomposite for wearable electrochemical–physiological hybrid biosensors has been successfully demonstrated in a recent study [189]. In addition, graphene-based nanomaterials are of particular importance as nanocarriers in the targeted administration of drugs, and for this purpose, their use in vaccines is envisaged [190].

## Figures and Tables

**Table 2:** Various GO-based biosensors for BC.

Electrode	Detection Technique	Target	LR	LOD	Ref.
GO/P3ABA/2D-MoSe_2_/ AuNPs	DPV	CA 15-3 miRNA-21	-	0.14 U.mL^−1^ 1.2 fM	[102]
GO/MCH ^a^/DNA/AuNPs/ SF ^b^/GCE	EIS	BRCA1	10^–16^–10^–8^ M	3.3 × 10^–17^ M	[103]
GO/GNR ^c^/GCE	DPV	miRNA-155	2 fM–8 pM	0.6 fM	[104]
CGO/Au-Pt BNPs/FTO	DPV	MUC1	1 fM–100 nM	0.79 fM	[105]
GO/GNR/GCE	EIS	miRNA-199a-5p	15 Fm–148 pM	4.5 fM	[106]
GO/AuNPs/PEDOT/FTO	DPV	MUC1	3.13 aM–31.25 nM	0.031 fM	[107]
GO/DNA/AuNPs/GCE	CA	HER2	-	0.23 nM	[108]
Apt/GO/AuNPs/GCE	DPV	MCF-7 cells	10–10^5^ cells.mL^−1^	8 cells.mL^−1^	[109]
GO/AuNPs/PtFe alloy/thionine/GCE	DPV	MCF-7 cells	100–5 × 10^7^cells.mL^−1^	38 cells.mL^−1^	[110]
GO-IL-PGEs ^d^	DPV	BRCA1	-	251 nM	[87]
GO/AuNPs/MgO nanoflower	DPV	miRNA-21	0.1–100 fM	0.05 fM	[111]
GO/ITO	-	CA 15-3	-	0.1 U/mL	[112]
MGO/Avastin/Au electrode	DPV	VEGF	31.25–2000 pg.mL^−1^	31.25 pg.mL^−1^	[113]
GO/IL/AuNPs/GCE	DPV	CD44	5 fg.mL^−1^–50 μg.mL^−1^	2 fg.mL^−1^	[114]

a: 6-Mercapto-1-hexanol, b: silk fibroin nanofibers, c: gold nanorods, d: pencil graphite electrodes.

**Table 3:** Various rGO-based biosensors for the diagnosis of BC.

Electrode	Detection Technique	Target	LR	LOD	Ref.
rGO/SWCNT/AuNPs/GCE	EIS	HER2	0.1 pg.mL^−1^–1 ng.mL^−1^	50 fg.mL^−1^	[139]
rGO/P2ABA ^a^/AuNPs/ SPCE ^b^	DPV	miRNA-155 miRNA-21 miRNA-16	1 fM to 10 nM 1 fM to 10 nM 1 fM to 10 nM	0.98 fM 3.58 fM 0.25 fM	[140]
rGO/anti-CEA/GCE	EIS	CEA	0.1–5 ng.mL^−1^	0.05 ng.mL^−1^	[141]
rGO/APTES ^c^/MoO_3_/ITO	DPV	HER2	0.001−500 ng.mL^−1^	0.001 ng.mL^−1^	[142]
rGO/CuS/SPGE	DPV	CA15-3	1–150 U.mL^−1^	0.3 U.mL^−1^	[143]
rGO/AuNPs/GCE	EIS	BRCA1	3 × 10^−20^–10^−12^ M 10^−12^–10^−7^ M	10^−20^ M	[144]
rGO/DNA/PANHS ^d^/GCE	EIS	BRCA1	10^−18^–10^−10^ M	3.5 × 10^−19^ M	[145]
rGO/AuNPs/PE	DPV	miRNA-21 miRNA-155	37.5 nM–150 nM 33.8 nM–135.3 nM	12 nM 25.7 nM	[2]
rGO/TiP-Cd^2 + e^-SA ^f^/Au NPs/GCE	SWV	miRNA-21	1 aM–10 pM	0.76 aM	[146]
rGO/trimetallic AuPtPd NPs/GCE	CA	H_2_O_2_ from MDA-MB-231 cells	0.005–6.5 mM	2 nM	[147]
rGO/Au-Pd NPs/GCE	CA	H_2_O_2_ from BC cells	0.005–3500 μM	0.004 μM	[148]
rGO/CS ^g^/AuNPs/GCE	EIS	MCF-7 cells	10–10^6^ cells.mL^−1^	4 cells.mL^−1^	[149]
rGO/TiO_2_ nanotube/MUC1 Apt.	EIS	MCF-7 cells	10^3^–10^7^ cells.mL^−1^	40 cells.mL^−1^	[150]
rGO/PP3CA ^h^/GCE	DPV EIS	BRCA1	10^−14^–10^−8^M	3 × 10^−15^ M	[151]
rGO/Au NPs/CuO NPs/GCE	DPV	MCF-7 cells	50–10^4^ cells.mL^−1^	27 cells.mL^−1^	[152]
rGO/MoS_2_/Fe_3_O_4_ NPs/GCE	DPV	MCF-7 cells	15–45 cells.mL^−1^	6 cells.mL^−1^	[153]
3D-rGO/PANI/GCE	DPV	BRCA1	10^−15^–10^−7^ M	3.01 × 10^−16^ M	[154]
rGO/Au NPs/PDA ^i^/MSNs ^j^/ GCE	SWV	CA 15-3	0.002–125 U/mL	0.002 U/mL	[155]
rGO/Au NPs/GCE	ACV	MUC1	1 pM–1 μM	0.25 pM	[156]
rGO/Au NPs	DPV	miRNA-21	10^−12^–10^−4^ M	1 pM	[157]
rGO/Ag NPs/Au NPs	CA	CA 15-3	15–125 U/mL	-	[158]
rGO/Ag NPs/TiO_2_	CA	CA 15-3	0.1–300 U/mL	0.07 U/mL	[159]
rGO/ZnMn_2_O_4_	CA	H_2_O_2_ from MCF-7	0.03–6000 μM	0.012 μM	[160]
rGO/CS/GCE	DPV	HER2	0.5–2 ng.mL^−1^ 2–75 ng.mL^−1^	0.22 ng.mL^−1^	[161]

a: Poly(2-amino-benzylamine), b: screen-printed carbon electrode, c: 3 aminopropyltriethoxysilane, d: (1-pyrenebutyric acid)-Nhydroxysuccinimide ester, e: Cd^2+^ functionalized titanium phosphate nanosphere, f: Streptavidin protein, g: Chitosan, h: iPolyPyrrole-3-carboxylic acid, i: Polydopamine, j: mesoporous silica nanoparticles.

**Table 4:** Various GQD-based biosensors for the diagnosis of BC.

Electrode	Detection Technique	Target	LR	LOD	Ref.
NGQDs/PHA-L/SPE	LSV	MCF-7 cells	5–10^6^ cells.mL^−1^	1 cells.mL^−1^	[184]
GQDs/CysA ^a^/Au NSs/ BSA ^b^/GCE	SWV	CA 15-3	0.3–1 U/mL and 2–250 U/mL	0.011 U/mL	[185]
GQDs/AuNPs/GO/SPCE	SWV	miRNA-21 miRNA-155 miRNA-210	0.001–1000 pM 0.001–1000 pM 0.001–1000 pM	0.04 fM 0.33 fM 0.28 fM	[186]
GQD/Ag@Au core–shell	CA	miRNA-21	5 pM–5 mM	-	[187]

a: Cysteamine, b: bovine serum albumin.

## Data Availability

Not applicable.
